# Knowledge Gaps and Emerging Research Areas in Intrauterine Growth Restriction-Associated Brain Injury

**DOI:** 10.3389/fendo.2019.00188

**Published:** 2019-03-29

**Authors:** Bobbi Fleiss, Flora Wong, Fiona Brownfoot, Isabelle K. Shearer, Olivier Baud, David W. Walker, Pierre Gressens, Mary Tolcos

**Affiliations:** ^1^School of Health and Biomedical Sciences, RMIT University, Bundoora, VIC, Australia; ^2^NeuroDiderot, INSERM, Université Paris Diderot, Sorbonne Paris Cité, Paris, France; ^3^Centre for the Developing Brain, School of Biomedical Engineering and Imaging Sciences, King's College London, St Thomas' Hospital, London, United Kingdom; ^4^The Ritchie Centre, Hudson Institute of Medical Research, Clayton, VIC, Australia; ^5^Department of Paediatrics, Monash University, Clayton, VIC, Australia; ^6^Monash Newborn, Monash Children's Hospital, Clayton, VIC, Australia; ^7^Translational Obstetrics Group, Department of Obstetrics and Gynaecology, Mercy Hospital for Women, University of Melbourne, Heidelberg, VIC, Australia; ^8^Division of Neonatal Intensive Care, University Hospitals of Geneva, Children's Hospital, University of Geneva, Geneva, Switzerland; ^9^PremUP, Paris, France

**Keywords:** growth restriction, neurobiology and brain physiology, brain development, neuroprotection, neuroinflammation

## Abstract

Intrauterine growth restriction (IUGR) is a complex global healthcare issue. Concerted research and clinical efforts have improved our knowledge of the neurodevelopmental sequelae of IUGR which has raised the profile of this complex problem. Nevertheless, there is still a lack of therapies to prevent the substantial rates of fetal demise or the constellation of permanent neurological deficits that arise from IUGR. The purpose of this article is to highlight the clinical and translational gaps in our knowledge that hamper our collective efforts to improve the neurological sequelae of IUGR. Also, we draw attention to cutting-edge tools and techniques that can provide novel insights into this disorder, and technologies that offer the potential for better drug design and delivery. We cover topics including: how we can improve our use of crib-side monitoring options, what we still need to know about inflammation in IUGR, the necessity for more human post-mortem studies, lessons from improved integrated histology-imaging analyses regarding the cell-specific nature of magnetic resonance imaging (MRI) signals, options to improve risk stratification with genomic analysis, and treatments mediated by nanoparticle delivery which are designed to modify specific cell functions.

## Introduction

Intrauterine (or fetal) growth restriction (IUGR, FGR) is caused by a heterogeneous set of maternal and fetal clinical pathologies. Stillbirth, neonatal mortality and poor neurological, and cardiovascular outcomes are all too common consequences of IUGR. We do not have the clinical tests to reliably predict the onset of IUGR, or even to reliably detect it, when present in late gestation. A diagnosis of IUGR is simply the indication that the infant has a birth weight below their genetically predetermined potential, with no etiological meaning. Our understanding of the clinical progression of IUGR including the poor neurodevelopmental outcomes has increased due to improved imaging techniques, such as Doppler velocimetry and magnetic resonance imaging (MRI). Nevertheless, as a research community, we are still striving to understand the pathological mechanisms leading to the various subtypes of IUGR (discussed below). Increase of this knowledge is crucial for tailoring therapies to prevent or treat IUGR and, in particular to reduce brain injury. Highlighting the need for a continuing collective research efforts is that, depending on the specific definition of IUGR applied (discussed below), the incidence of IUGR is between 3 and 9% of pregnancies in high resource settings, but horrifically, in low-resource settings, the rates are as high as 30% of pregnancies (1).

The purpose of our review, written in a narrative form [as defined in McGaghie (2)], is to highlight the gaps in our clinical and translational knowledge, and the weaknesses of our research methods that hamper our collective efforts to improve the neurological outcomes of those infants diagnosed with IUGR. Our opinions and interests are diverse—as authors we include clinicians working as obstetricians, neonatologists, and neonatal neurologists, plus researchers with expertise in fetal physiology and developmental neurobiology. We will focus on new cutting-edge tools and techniques that might provide novel insights into this disorder of fetal growth, and on technologies that offer the potential for better drug design and delivery. We will cover topics including maximizing the benefit of data from scarce human post-mortem tissues, lessons from advanced histological analyses regarding the cell-specific nature of MRI signals, options to improve risk stratification with genomic analysis, and new treatments based on delivery of nanoparticles designed to modify cell-specific functions.

## What is IUGR?

The issue of an optimal definition for IUGR has been called “one of the most common, controversial, and complex problems in obstetrics” (3). Simply, IUGR is usually recognized when an infant appears to have failed to grow to its expected size—based on it genetically pre-determined potential. Most surprisingly though, there is no universally applied definition of what threshold of body weight clearly defines an infant as IUGR, with birth weights at or below the 10th or the 3rd centile, and concomitant changes in placental blood flow and gestational age all taken into account with varying frequencies across health care centers even within countries. An advance in bringing a universally recognized definition to the field was made with the recent publication of a Delphi procedure resulting in an agreed definition of IUGR (4). However, although a Delphi procedure is a well-established method for finding consensus, only 45 participants from across the entire world took part; 54% of these being from Europe, encompassing 37 member states with various population risk factors and health care paradigms. In addition, Asia and Australia were grouped as a single entity with only 10 opinions received from these diverse areas of the world. The Delphi procedure presented clinical variables and outcomes already known to be associated with IUGR and asked the participants to rank or stratify the importance of these. The end result was that somatic growth indices, umbilical artery pulsatility index, and absent end-diastolic flow were the favored diagnostic criteria. At this point we would like to highlight there is no pathophysiological meaning within the term IUGR (or FGR) as IUGR ultimately is a symptom, like the diagnosis of microcephaly (i.e., brain growth at or below two standard deviations from the mean). The term IUGR (or FGR) is as useful for understanding the disorder as if we used the term PRBS (poorly regulated blood sugar) in place of diabetes. The Delphi procedure offers no clarification on whether it is possible to stratify patients based on the underlying cause of their IUGR, although admittedly, this was not its purpose. However, we need to revaluate the criteria currently used to determine IUGR, (e.g., somatic growth, umbilical blood flow) some of which might well-reflect compensation and adaptation to an underlying, unidentified causal mechanism. Additional measurements could include blood-based biochemical measures of inflammation, placentally-derived biomarkers, postnatal blood pressure and urine parameters, and additional biometrics such as skin folds, and skeletal phenotype. Meta-analysis of clinical data banks, together with studies on stored chorionic villi and maternal blood retained after routine clinical testing, and (albeit, complicated) the recruitment of further large prospective cohorts—could provide us with biochemical parameters that closely reflect the underlying causes of reduced fetal growth, and how these stratify with risk across the lifespan.

How we name a disease has a significant bearing on how we then think about it, and ultimately, how we model and aim to treat it, so these issues of universal nomenclature are important; see commentaries by Dammann et al. (5), and McIntyre et al. (6) for the importance of classifying known causes, and not symptomatic outcomes. A recent summary of the definition(s) of IUGR as they have evolved over the past 30 years highlights three facts: (1) that a significant number of clinical studies on IUGR (11%) gave no definition of IUGR; (2) that ultrasound measurements of fetal biometrics, but not Doppler measurements of placental and/or fetal brain blood flows, have been increasingly used to define IUGR, and; (3) that overall the primary, consistently used characteristic of IUGR diagnosis is birth weight (7). This last observation highlights that we are missing opportunities to identify the early prenatal events that lead to IUGR, and therefore of developing our approaches to increase the efficacy of interventions, and the opportunity to prevent, rather than repair brain damage and therefore to improve neurological outcomes for these infants via early identification. A further confounder in diagnosing IUGR is the lack of accurate gestational dating, as in many countries (including the USA) routine ultrasound dating scans are not available and estimates based on last menstrual period are generally unreliable.

Maternal and fetal genetic factors are responsible for >50% of the variance in birth weight (8). As such, is it critical to separate healthy infants that are small for gestational age (SGA) due to inherent genetic factors from those with reduced growth that are at risk of sustaining neurological injury and metabolic dysfunction. Personalized growth charts, based on maternal and fetal characteristics (such as age, body weight, fetal sex, and ethnicity), have improved our ability to differentiate IUGR and SGA infants [for a commentary see (9)]. While correcting for maternal parameters seems appropriate, perhaps correcting for ethnicity might normalize a socially disadvantaged group with higher rates of growth restriction and poor obstetric outcome. This was perhaps best reflected in INTERGORWTH 21 which recruited over 20,000 pregnant women from numerous countries around the world in regions where the health and nutrition of the mother were met and antenatal care was adequate, and they demonstrated that the growth parameters were similar regardless of ethnicity.

IUGR is also classified as symmetric or asymmetric IUGR (**Figure 2**), dependent on the ratio of the head circumference to the abdominal circumference, which is increased in asymmetric IUGR. This categorization is based on the idea of brain sparing arising (typically) from late-onset IUGR. Early-onset IUGR (before 32 weeks post-conceptional age [PCA]) is more often associated with the symmetrical form of IUGR, and late-onset IUGR (after 31 + 6 weeks PCA) is more often associated with asymmetric IUGR. For further details on brain sparing and a discussion on the consequences and relationship between adaptive to maladaptive (compensatory vs. decompensatory) processes, see the section on “Causes of IUGR,” below. There is also a third phenotype of IUGR reflecting the accumulated effects of early *and* late IUGR risk factors (10). This mixed phenotype is observed predominantly in pregnancies complicated by peri-conceptual malnutrition, and then by placental dysfunction later in pregnancy. These infants are suggested to present with symmetric growth, but with severe signs of malnutrition, such as high numbers of scapula skin folds (10).

## What are the Outcomes Associated With IUGR?

IUGR fetuses are at increased risk of stillbirth, fetal compromise, early neonatal death, and neonatal morbidity (11). A vast literature including many works from Winder et al. has demonstrated that the availability of physiological resources that support growth *in utero*, which include not only maternal nutritional status (12) but also placental size, shape, and metabolic efficiency, have effects that continue to have an impact on health throughout childhood and adult life (13, 14). Indeed, IUGR-born infants are prone to a range of health problems, including increased risk of cardiovascular diseases and neurodevelopmental disorders (15, 16).

It has been difficult to compile outcome data from the many studies on IUGR, because in addition to the fact that IUGR includes an inconsistent, heterogeneous set of clinical characteristics and underlying etiologies, postnatal data reporting includes further inconsistencies in patient selection and outcome parameters. To enable future trials to measure similar meaningful outcomes, the Core Outcome Set for GROwth restriction: deVeloping Endpoints (COSGROVE) consortium is developing two core outcome sets—one for prevention and the other for treatment of IUGR (17). These guidelines will ensure the collection and reporting of a minimum dataset, agreed by stakeholder consensus that will reduce inconsistencies in the reporting of outcomes across relevant trials. For comprehensive reviews on IUGR-related brain injury, including specifics related to the type and severity of IUGR and outcomes, we refer the reader to Miller et al. (18), Tolcos et al. (19), and Gilchrist et al. (20). Over all, infants that were born IUGR have a significantly increased risk of motor and sensory neurodevelopmental deficits, cognitive and learning impairments, and cerebral palsy (see papers above). In particular, in near-term and term infants the rates of cerebral palsy are higher in IUGR infants (16.5%) than the rates in infants exposed to birth asphyxia (8.5%) or inflammation (4.8%) (21). A common comorbidity of IUGR is preterm birth, and this confounds our understanding of the specific effects of IUGR on brain development and function (discussed below). In addition, the risk of delivering an IUGR baby is higher for woman with chronic hypertension, pre-eclampsia, low socioeconomic status, overt diabetes, anemia, gestational diabetes mellitus, low pre-pregnancy body mass index, or hypothyroidism (22). Whether each of these risk factors leads to a specific phenotype of outcome for the IUGR infant is still a matter that requires considerable study. A number of these risk factors and their causal role in IUGR is discussed further below.

## What are the Causes of IUGR?

**Figure 1:**
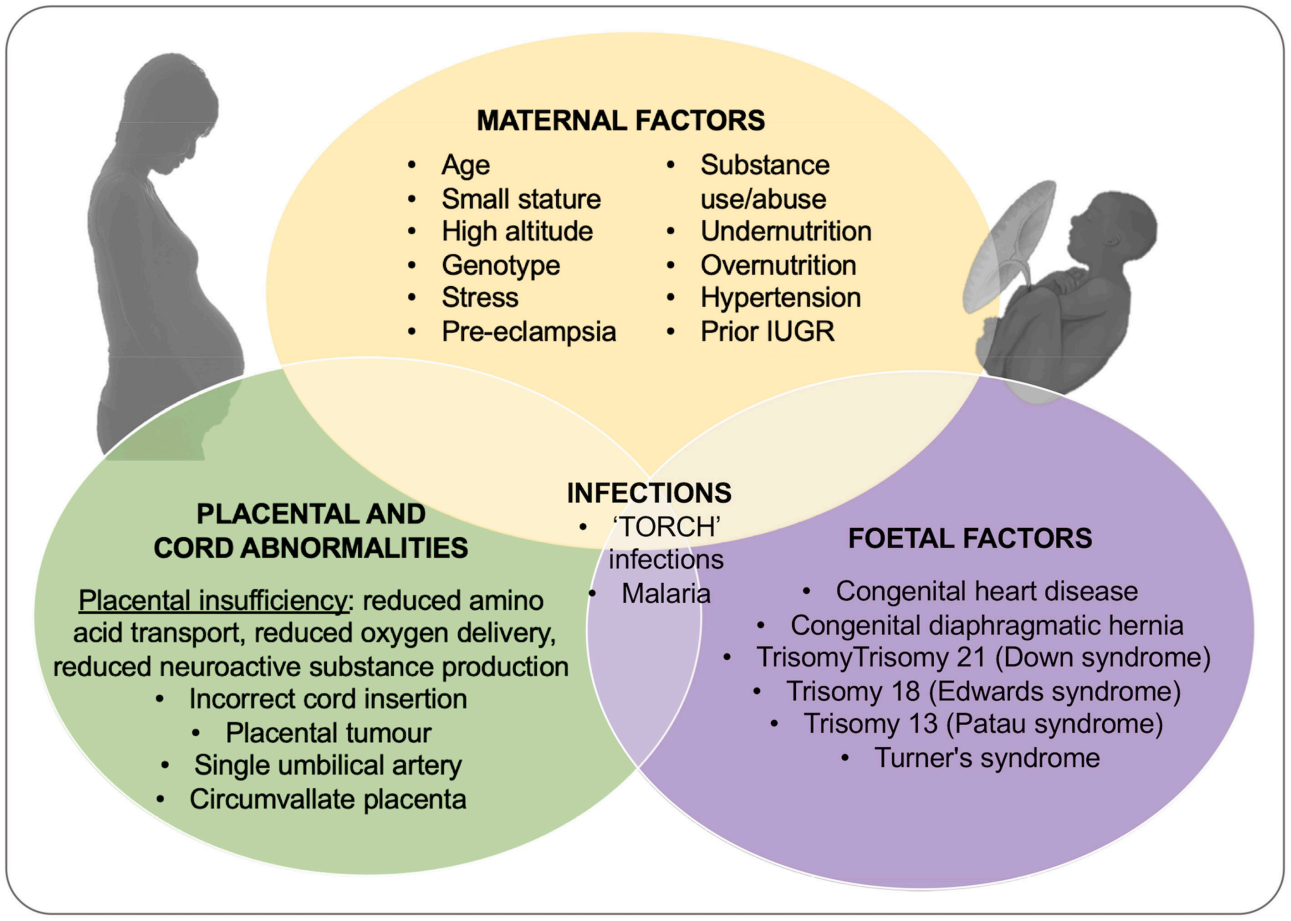
Outline of the causes of IUGR including contributions from maternal, placental and umbilical cord, and fetal dysfunction or injury. Adapted from Vijayaselvi and Cherian (22) and Gaccioli and Lager (23).

The primary cause of IUGR is widely considered to be placental insufficiency; i.e., inability of the placenta to adequately support fetal growth. However, the causes of placental insufficiency are many and over-lapping, and include constricted spiral arteries and increased coagulation leading to fetal hypoxia (as in maternal hypertension), and inappropriate substrate availability due to maternal under-nutrition or over-nutrition (22, 23) (see Figure 1). For the purposes of this review we won't discuss in detail the relationship between IUGR and maternal drug use (alcohol, tobacco, cocaine etc) or the association with infectious agents, the so-called “TORCH” infections; Toxoplasmosis, Other (syphilis, hepatitis B, varicella-zoster virus, human immunodeficiency virus [HIV], parvovirus B19, enteroviruses, lymphocytic choriomeningitic virus etc.), Rubella, Cytomegalovirus, and Herpes simplex virus. However, these infections and exposure to environmental toxins often lead to a complex constellation of outcomes (microcephaly, facial abnormalities, intracranial calcifications, rash, jaundice, hepatosplenomegaly, elevated transaminase concentrations, and thrombocytopenia) which, although low in incidence in high-income settings (24), are significant in low resource settings (25).

### Late vs. Early Onset IUGR and the “*Head-Sparing*” Effect

**Figure 2:**
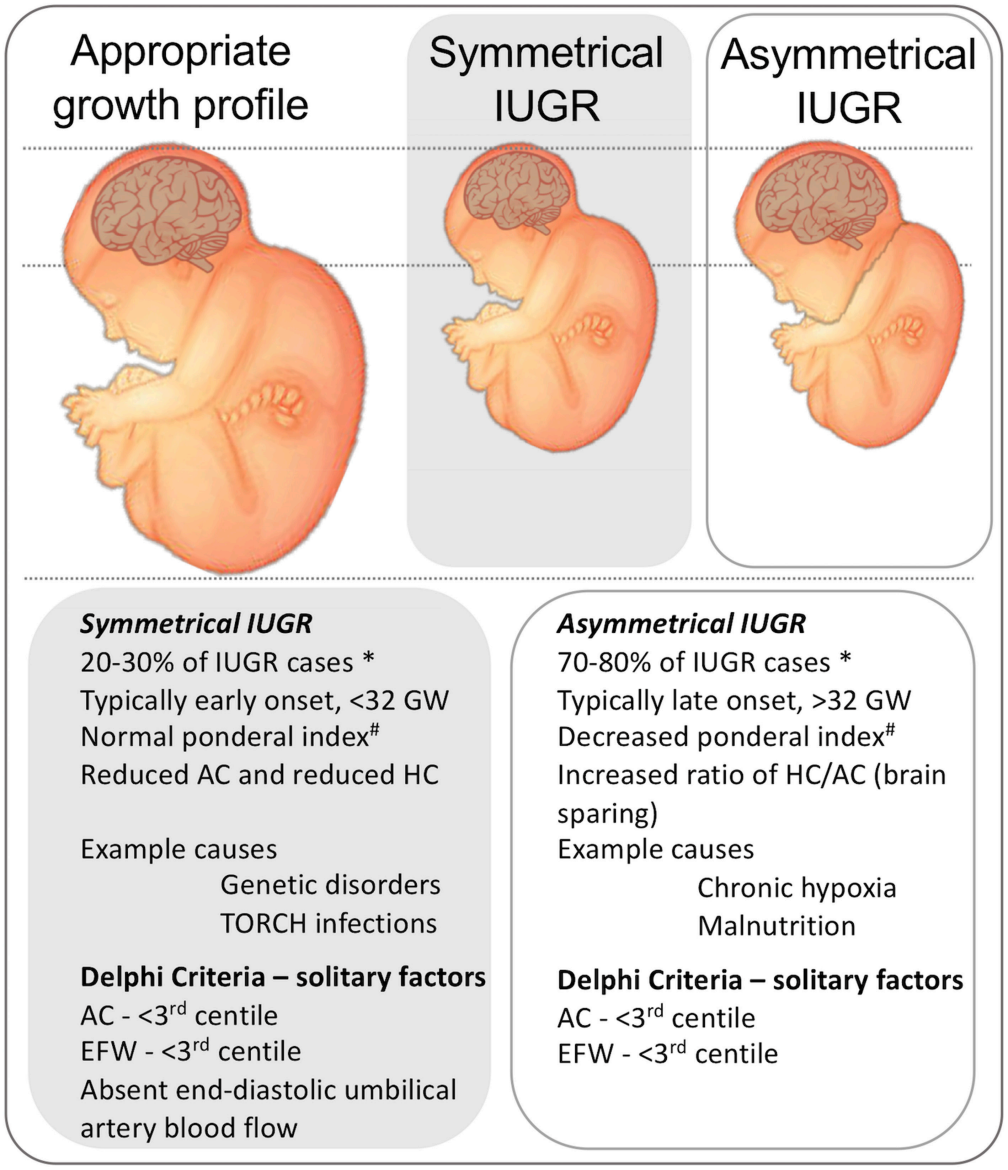
Representation of the physical presentation of symmetrical and asymmetrical IUGR and a short list of clinical characteristics and causes. ^*^ note that incidence data are from high-resource settings. A third phenotype is proposed in low-resource settings, that includes characteristics of malnutrition and late gestation placental insufficiency (10), not shown. # Ponderal index, (birth weight (g)/length(cm)^3^ × 100). HC, head circumference. AC, Abdominal circumference. GW, gestational weeks. AC, abdominal circumference. EFW, Estimated fetal weight. Delphi criteria from Gordijn et al. (4).

In addition to linking IUGR to specific pathological processes, as discussed above we also classify IUGR as symmetric or asymmetric (Figure 2), dependent on the ratio of the head circumference to the abdominal circumference of the infant. Asymmetric IUGR is the result of “brain sparing,” a process whereby brain growth is less affected than body growth due to the redistribution of cardiac output. While brain sparing does not completely prevent the damaging effects of IUGR on brain development (26, 27), it is nonetheless associated with better neurological outcomes than when brain sparing does not occur (28). It is also worth noting that mortality is higher in IUGR with symmetric growth even after adjusting for possible cofounding factors (26). Brain sparing can be detected prenatally based on the Doppler pulsatility index (PI) in the middle cerebral artery (MCA); PI is reduced by the decreased cerebral resistance which allows a greater fraction of the cardiac output to perfuse the brain. However, this compensatory process of blood distribution can become “decompensatory” because the increase of brain blood flow and blood volume themselves become damaging (29). Understanding when and how the alteration of relative cerebral blood flow is switched from a compensatory to decompensatory response is clearly important for devising the most appropriate interventions and therapies. It is worth noting that brain sparing does not reduce the consequences of IUGR on later health, such as increased adiposity, diabetes, and cardiovascular risks, etc [reviewed in Sehgal et al. (30) and Devaskar and Chu (31)].

Maternal risk factors, especially diabetes, high body-mass-index and hypertension, are relevant risk factors for IUGR across both high- and low-income settings. This blurring of income-related demarcations is driven in part by changes in diet and lifestyle, such as adoption of a “western-style” diet and changes in the nature of “work” with more people employed in sedentary activities in urban centers across continents. An illustration of this is the increased risk for an IUGR infant when a woman is diabetic; in Africa the rate of diabetes in adults 25–64 years of age is at 15%, in India 9%, and in Australia 5% (WHO, heath-topics, 2018). An increased body-mass-index is also a major risk factor for gestational diabetes that also associates with poor fetal outcomes. The effects of diabetes on the placenta occur irrespective of the cause of diabetes and the careful management of blood sugar, and include thickening of the trophoblast basement membrane (32), which impairs oxygen and nutrient delivery. Diabetes is associated with a higher release of placental cytokines such as leptin, tumor necrosis factor-α (TNF-α), and Interleukin-6 (IL-6) [see Pantham et al. (33)]. Over-nutrition in the (apparent) absence of co-morbidities is also associated with poor placental development/function and poor fetal outcomes [recently reviewed in Howell and Powell (34)], and this has also been demonstrated in experimental models, including sheep, mouse, rat, and rabbit (35–37).

An important link between maternal overnutrition, poor placental development, and poor brain development is inflammation. Obesity is a known driver of systemic inflammation and neuroinflammation (see microglial section, below) (38), and a precise regulation of the maternal and fetal immune system is required for proper placental function and fetal brain development. Of note, adipose tissue produces adipokines, including the pro-inflammatory factors TNF-*a*, IL-6, and MCP1 (macrophage chemotactic protein 1 also known as CCL2). This is considered to explain (at least, in part) why maternal obesity is associated with higher levels of circulating inflammatory mediators during pregnancy and dysregulated placental nutrient transport (39, 40); such systemic and placental inflammatory effects can be reproduced by over-nutrition in pregnant sheep, leading to IUGR (41, 42).

Preeclampsia increases the risks for IUGR 4-fold and is a leading cause of maternal and fetal death. Preeclampsia is a condition of vascular endothelial dysfunction and vasospasm that occurs after 20 weeks of gestation that has its origins in inadequate trophoblastic invasion of the uterine vascular bed, in particular, of the spiral arteries. World-wide, preeclampsia occurs in 8–10% of pregnancies and this rate is consistent across high, middle-, and low-income settings. The factors associated with the onset of preeclampsia include pregnancy at a young or advanced age, high maternal body-mass-index and psychosocial stress. Preeclampsia is usually associated with IUGR, but early onset preeclampsia may be associated with an enlarged placenta and over-large birth weight. While reports show there are differences in placental function and biomarkers between IUGR and preeclampsia, there is overlap between the outcome in these disorders and understanding the similarities and differences will be useful for managing maternal and fetal risk (43–47).

In low resource settings malnutrition is a leading causal factor in IUGR, and all too often these IUGR infants are also delivered preterm adding to the burden of mortality and neurodevelopmental injury (48, 49). Malnutrition is linked not only to economic factors, but also to cultural and social norms that include young maternal age, and repeated and closely spaced pregnancies. UNICEF estimates that in the most developed countries 7% of babies are of low birth weight (encompassing prematurity and IUGR predominantly), whereas in less developed, and the least developed countries, rates of low birth weight deliveries increase to 16.5 and 18.6%, respectively, representing more than 22 million babies annually. Although great improvements in health and well-being are being made, especially as part of the Millennium Development Goals, specific focus on how to overcome poor neurological outcomes for the infants born to these mothers is still greatly needed. For more details on IUGR in less well-developed countries please refer to these references (1, 10, 48–52). Trials of nutritional supplements (micronutrient-supplemented protein, balanced calorie supplements etc.) in women with an IUGR pregnancy identified in the 2nd trimester have failed to improve outcomes, and overall the literature supports pre- and peri-conceptional nutrition as a major determinant of fetal development and pregnancy outcome [reviewed in Liberato et al. (53)].

## What are the Primary Neuropathological Processes in IUGR?

### Placental Dysfunction

Early-onset IUGR is associated with high impedance uteroplacental perfusion and elevated umbilical artery blood flow resistance as measured by Doppler ultrasound [reviewed in detail by Dall'Asta et al. (54)]. Late-onset IUGR is more common, occurring in 70–80% of IUGR cases and the diagnosis and monitoring of late-onset IUGR has been recently reviewed by Figueras et al. (55). Overt placental pathology may be mild, or even absent in late-onset IUGR, and the Doppler parameters of umbilical artery blood flow may even be normal, but changes in brain blood flow dynamics, such as fetal middle cerebral artery impedance, may be reported indicating compensatory changes to cardiac output indicative of established IUGR. In addition to changes in uterine artery blood flow indicating high vascular resistance, placental studies reveal the important contribution of placental infarcts, and changes in placental amino acid and micro-nutrient transporters, and pathways for growth factor production, further support the concept of “functional” placental insufficiency of IUGR (56, 57). Based on these human studies, animal models of IUGR have been created that recapitulate aspects of the human condition (discussed below), and by and large demonstrate the link between the deprivation of oxygen, glucose, amino acid, and growth factors to the slowing of fetal growth. In addition to this it is now apparent that the abnormal change in placental metabolism results in increased production of reactive oxygen species (ROS), aberrant activation of the complement cascade, re-programming of microglial phenotype (see below), and changes in the trajectory of brain maturation that include dysregulated neural cell proliferation, slower maturation of oligodendrocytes resulting in hypomyelination, and increased programmed (apoptotic) cell death (58–61). We refer the reader to publications describing the mechanisms underpinning the neuropathology of IUGR for more details on these processes (19, 62, 63).

In addition to the somewhat obvious link between the placenta as a source of cytotoxicity, there is the possibility that growth promoting cytokines and neurotrophic growth factors are altered with placental insufficiency. In addition to somatic growth factors such as IGF1, the placenta is a source of pregnene and androgen steroids that directly, or via metabolites such as allopregnanolone, promote, and protect fetal brain development by promoting fetal sleep and quiescence [(64);and references within (65)]. It has been hypothesized the allopregnanolone is an endogenous neuroprotectant, via its effect of global inhibition of CNS activity, that protects against antenatal brain injury (66–68).This field of steroid research has given rise to the notion that there is a “placenta-brain axis” that reflects the co-ordinate development of the fetal adrenal gland, liver, and steroid metabolizing functions of the placenta (69, 70). Neurosteroids derived from progesterone such as allopregnanolone interact with GABA-A receptors and increase central nervous system (CNS) inhibition (71, 72). In uncomplicated pregnancies there are high levels of neurosteroids such as allopregnanolone in the fetal brain immediately before birth, but these levels fall rapidly with removal of the placenta because they are cleared quickly from the circulation, and have a half-life of only minutes (73). In the placenta of infants born preterm the activity of these neurosteroidogenic pathways is reduced, raising the possibility that the prenatal and postnatal paucity of allopregnanolone might affect brain development in these infants (74). In the serum of pregnant women carrying an IUGR fetus, levels of allopregnanolone are lower (75), and in animal models of IUGR the neurosteroid systems (genes and proteins) are lower in the fetal brain (76, 77). Direct inhibition of allopregnanolone production during development causes brain injury (78, 79) and lasting behavioral deficits (80). It is thus reasonable to suggest that progesterone, and even allopregnanolone, should be replaced in preterm and IUGR infants to improve neurological outcomes (81). For example, Ganaxolone is a synthetic analog of allopregnanolone that has a long half-life, has shown promise as a seizure therapy in adults and children refractory to anti-seizure treatments, and in animal studies does not alter fetal viability, neonatal growth, and is without teratogenic or genotoxic effects [reviewed in Hirst et al. (81)]. Ganaxolone therapy should (like all drugs) be trialed in large animal models (powered for sex and with long term outcome) of the various types of IUGR that can now be modeled—for example, by delayed onset chronic hypoxia such as the single artery ligation (SUAL) sheep model (82); early onset placental insufficiency in piglets—(60); maternal overnutrition before and during pregnancy in the sheep—(83). An important layer of safety data that needs to be obtained is the specific interaction that early treatment with ganaxolone might have on the developmental switch of GABA receptors from excitatory to inhibitory (84). This event is predicted to be before 26 weeks' gestation in humans and it might be expected that before this time that ganaxolone would induce, not supress, neuronal activity.

Of the amino acids transported and metabolized by the placenta, tryptophan has a special significance (85). It is an essential amino acid, but protein synthesis accounts for only a minor part of its fate, the greater part being committed to the kynurenine and serotonin pathways. Conversion of tryptophan to kynurenine via indoleamine 2,3-dioxygenase (IDO) in early pregnancy may be important for immune suppression and acceptance of the conceptus as an allograft (86). Later in pregnancy the synthesis of kynurenine may be more important because it is the precursor of kynurenic acid, a physiological glutamate receptor antagonist, which acts as a neuroprotectant (87). Both gene and protein expression of IDO and tryptophan 2,3-dioxygenase (the second enzyme in the kynurenine to tryptophan conversion) are significantly lower in IUGR-affected placentas compared with controls [reviewed by, (88)]. IDO is an oxygenase, and its activity is downregulated in reduced oxygen conditions; demonstrated in *ex vivo* first and third trimester human placental explants exposed to lower oxygen (5–8% O_2_) or higher oxygen (20% O_2_) conditions. Exposure to lower oxygen levels reduced IDO mRNA and protein expression, and other kynurenine pathway enzymes and kynurenine output was also significantly reduced (88). Inflammatory mediators, such as TNF-α, IL-1β, and interferon-gamma, induce IDO expression thereby increasing tryptophan degradation to kynurenine, but with the result that pro-oxidant (e.g., 3OH-anthranilic acid) and glutamate agonist metabolites (e.g., quinolinic acid) are produced (85). The impact of IUGR on the placental metabolism of tryptophan is not fully understood, but it can be seen from the above that placental insufficiency could have a significant impact on the fetal brain via alterations in the placental degradation of tryptophan.

Tryptophan is also the precursor for the synthesis of serotonin (5-HT). Abnormal levels of brain 5HT have been linked to neurodevelopmental disorders such as autism spectrum disorder (ASD) (89), but when these abnormalities arise in humans (i.e., antenatally or postnatally?) is unclear. There is evidence from mouse pregnancies that placental 5-HT has an important role in early fetal brain development, in that the 5-HT needed for early forebrain development initially comes from the placenta (90). In early neurodevelopment 5-HT functions to regulate a number of key processes, including cell proliferation and neuronal differentiation, migration, and synaptogenesis (89), and experiments in the mouse clearly show the free entry of 5-HT into the immature brain. However, by late gestation, there is a decrease of placental 5-HT synthesis in humans and mice as the raphe nuclei in the midbrain become competent and 5HT axons reach the forebrain (90). This co-ordinate change of 5-HT synthesis between the placenta and brain really does suggest the presence of a “placenta-brain axis” which should be investigated more fully in experimental settings using where IUGR, placental insufficiency, and preterm birth can be modeled in animals with more relevance to human pregnancy. Specifically, an issue with traditional mouse studies is that the fetus is delivered at a stage of brain development equivalent to the start of the second trimester in the human; this makes studies of the last trimester fetal-placental axis impossible. However, peripherally synthesized 5-HT does not freely cross the blood-brain barrier in more fully developed (i.e., adult) brains (91). Goeden et al. (92) have also demonstrated the effects of mild maternal inflammation on placental tryptophan catabolism to 5-HT. Their findings suggest that maternal inflammation during human pregnancy may lead to increased 5-HT synthesis in the placenta and output to the fetus, resulting in abnormal serotonergic axon outgrowth into the developing forebrain.

It is therefore evident that chronic placental hypoxia and inflammation affect the catabolism of tryptophan in the placenta. It is suggested that IDO may act as a “sink” for superoxide, since IDO is known to utilize the superoxide anion as well as molecular oxygen for its oxygenase activity (93). A decrease in IDO expression as a result of hypoxia may therefore lead to decreased clearance of superoxide and an inflammatory response, potentially increasing placental 5-HT synthesis, with consequences for brain growth (94). Alternatively, decreased kynurenine synthesis as a result of hypoxia may shift the tryptophan catabolism pathway in favor of 5-HT synthesis. Clearly, the full effects of IUGR and placental hypoxia on placental tryptophan catabolism are largely unknown but likely to be important for setting the chemical environment in the IUGR brain, and determining vulnerability to damage arising from hypoxia, oxidative stress, or inflammation.

### Inflammation and Neuroinflammation

The term inflammation is used to describe the production of cytokines, chemokines, reactive oxygen species, and secondary messengers, together with the paracrine and autocrine effects of these factors. In the CNS, microglia and astrocytes are the primary drivers of inflammation, i.e., neuroinflammation. Both systemic inflammation and neuroinflammation play a central role in the pathophysiology of various forms of perinatal brain damage, as shown by observations from both animal models and the human neonate (95–97). The levels of circulating cytokines in neonates born after IUGR are significantly increased on postnatal days 7 and 14 compared to levels measured in neonates without IUGR (98–100). This postnatal systemic pro-inflammatory state following IUGR could be, at least in part, responsible for the frank brain damage and neurodevelopmental impairments detected in childhood in these individuals. Indeed, at the heart of the vulnerability of the immature brain lies the systemic up-regulation of pro-inflammatory cytokines and the diffuse activation of cerebral microglia, the mediators of brain inflammation (95). The activation of microglia and astrocytes occurs via inflammatory signals coming from the systemic circulation via receptors on endothelial cells and the vagal nerve, and also by local pathogen- and damage-associated proteins (PAMPS and DAMPS, respectively) [reviewed in Carty and Bowie (101)]. In the context of IUGR, and in the absence of obvious pathogens, inflammation can come from at least two sources: (i) inflammation propagating from the placenta owing to the release of DAMPS due to tissue injury (see paragraph below), aberrant macrophage activation (102), and idiopathic villitis (103), and; (ii) from direct effects of hypoxia or other nutrient deprivation or intoxication on the brain (39–42).

Brain cell death, as a predicted consequence of hypoxia and other deprivations on the brain, would cause the release of DAMPS, such as HMGB1 (high mobility group box 1) that activate immune cells including microglia. Neuroinflammation, mediated by microglia, perturbs normal brain development directly by causing injury to cells such as maturing oligodendrocytes. In addition, an important set of developmental processes fulfilled by microglia is left undone when microglia are recruited to a neuroinflammatory response and this also damages the developing brain (see developmental dysfunction, below for further information on the role of microglia). A study of gene expression in microglia and oligodendrocytes in a model of protein restriction-induced IUGR has revealed a striking induction of inflammation-related genes in microglia accompanying the reduction in oligodendrocyte maturation and connectivity and functional deficits (59). This was the first comprehensive study to link protein-restriction with neuroinflammatory-associated brain injury, and in the future, we will look for similarities in related animal models, and undertake human post-mortem studies to look for these dysregulated pathways. We also point the reader toward two excellent reviews on the role of microglia/neuroinflammation in IUGR, one written by a team of experts in pre-clinical modeling and brain injury including observations of microglia and macro-gliosis in various animal models, Wixey et al. (104), and one from the perspective of experienced reproductive immunologists with a focus on maternal immune activation as a driver of microglial activation, Prins et al. (105).

It is worth noting that neuroinflammation (i.e., activated microglia) typically carries assumptions of completely maladaptive or damaging processes. However, less well-understood processes of protection, repair and regeneration are also mediated by micro- and macro-glia, which occur at specific times after injury or insult (106–109). Although there has not been sufficient study of microglial phenotypes in clinical or preclinical IUGR models, more knowledge on microglial activation (phenotypes and temporal regulation) will likely help the development of drugs and treatments that exploit and expand the reparative effects of microglia, while decreasing the negative effects of these cells. Harnessing microglia to regenerate the brain is an approach being applied in the field of multiple sclerosis and adult neurodegeneration (110–112), and it clearly has a place in neonatal neurology.

### Developmental Disturbance

The trajectory of brain development is altered by the presence of damaging stimuli, but also by the loss of cells, processes and factors that are important for brain building. As mentioned above, the causes and effects of IUGR include processes of inflammation, placental growth factor deprivation and hypoxia, which together affect the trophic actions of secreted factors such as serotonin, allopregnanolone, tryptophan, and IGF1/2. In addition, we wish to highlight that the “distraction” of microglia away from their normal physiological role in promoting proliferation, pathfinding, myelination and synaptogenesis will cause significant damage to the developmental trajectory of the brain [reviewed extensively in Prins et al. (105), Hagberg et al. (113), Pierre et al. (114) and Tay et al. (115)]

## Managing the Risks of Continuing Pregnancy vs. Preterm Birth—the Prenatal Care Team

As there are currently no effective medical interventions for IUGR, management consists of close surveillance aimed at determining the most appropriate time for delivery. The definition of “most appropriate time” is a question for which the prenatal team has few specific criteria, and balancing the risks of prematurity with the consequences of IUGR (including, stillbirth) remains a contentious issue. Clinical trials to determine the optimal time of delivery have focused on survival and immediate perinatal outcomes, and often lack long-term follow up or an exploration of what features of delivery result in optimal long-term outcomes and reduced neurodevelopmental complications. Recent randomized controlled trials (e.g., Growth Restriction Intervention Trial [GRIT]; Trial of Randomized Umbilical and Fetal Flow in Europe [TRUFFLE]) have helped to shed light on delivery parameters of IUGR babies that lead to improved long-term outcomes. Both studies had a primary outcome of neurodevelopmental delay at 2 years of age.

The GRIT study randomized women at 24–36 weeks PCA to early or delayed delivery and included patients when the clinician was in equipoise about whether they needed delivery. “A priori” parameters set to determine when patients would be delivered were not used in this study. This agnostic approach can be seen as a benefit, as retrospective analysis could shed light on novel parameters common to infants who did well, but it is also difficult, as the motivation for the clinician's decisions are not easy to describe or document, and may not have been adequately captured in the analysis. There was generally only a 4-day delivery interval delay between randomizing the participants to immediate vs. delayed delivery. The 2- and 7 year follow-ups did not show a difference in neurodevelopmental outcome between groups (discussed further below)

The TRUFFLE trial was performed to examine whether parameters set “a priori” for delivery are effective in optimizing delivery outcomes. Participants with IUGR diagnosed at 26–32 weeks PCA with an elevated umbilical artery pulsatility index were randomized to delivery based on: (1) reduced cardiotocography fetal heart rate short-term variation (STV-CTG); or (2) early ductus venosus (DV) changes via doppler; or (3) late ductus venosus changes. They found that more infants randomly assigned to delivery based on late changes in the ductus venosus (95%) were free of neurological impairment compared to those assigned to cardiotocography (85%), but this was accompanied by a non-significant increase in perinatal and infant mortality. They therefore concluded that delivery based on late changes in DV flow might reduce long-term neuro-impairment. When the actual criteria for delivery in these cohorts was dissected it became clear that the majority of patients in the delivery for DV changes were being delivered based on the safety net criteria of spontaneous decelerations in the fetal heart rate. When the two cohorts being delivered for DV changes were combined the neurodevelopmental outcomes at 2 years of age were more favorable compared to those delivered based on STV CTG changes. Therefore, optimal neurodevelopmental outcomes may result if delivery of very preterm patients is restricted until late changes arise in the DV with a caveat of delivery whenever the CTG is abnormal (116, 117).

Severe growth restriction at term is also associated with poor neurodevelopmental outcomes (118). The Disproportionate Intrauterine Growth Intervention Trial At Term (DIGITAT) assessed the impact of immediate delivery vs. expectant management in patients with growth-restricted fetuses at term. The follow-up at 2 years of age demonstrated similar neurodevelopmental gains. However, when assessed by gestation at birth, those in the lowest 2.3 percentile had significantly more neurodevelopmental compromise on an “ages and stages” questionnaire compared to those with a higher birth weight centile. Indeed, 43% of babies born with a birth weight centile below 2.3 had an abnormality on the their “ages and stages” questionnaire compared to 29% of those born at <10th centile and 13% of those with a birth weight greater than the 10th centile. This is consistent with a number of cohort studies demonstrating that low birth weight resulted in increased learning difficulties, defects in speech, neurological deficits, and behavioral problems (119).

## Brain Injury in the Preterm Born IUGR Infant—the Postnatal Care Team

The incidence of spontaneous preterm birth in pregnancies with severe IUGR is 2 to 3-fold greater than the incidence of pregnancies with appropriate fetal growth (120). In addition, antenatal care focuses heavily on fetal growth monitoring in order to identify pregnancies with poor growth that may benefit from a timely, planned preterm delivery to improve outcomes. However, the contribution of antenatal compromise vs. the postnatal complication of being born preterm, or potential interactions between the two, in contributing to the neurodevelopmental sequelae of IUGR is still unclear. This is important for the clinical decision on when to deliver, when IUGR is diagnosed antenatally.

In a large cohort study comparing more than 1,400 preterm IUGR infants with age-matched AGA controls from 25 to 32 weeks PCA, the incidence of severe intraventricular hemorrhage (IVH) in each gestational age group was similar, but prematurely born IUGR neonates had increased morbidity and mortality (121). On the other hand, preterm birth has been suggested to override the effects of IUGR *per se* on neurological outcomes (122, 123), with the impact most marked for births at the earlier gestational ages. In early-onset IUGR, the gestational age at delivery shows a consistent independent relationship with parameters of motor development with a maximum impact for infants delivered before 28 weeks PCA (124–126), independent of the severity of attrition of growth and the degree of cardiovascular and biophysical deterioration. In the GRIT study which randomly allocated women to early or delayed delivery in the presence of IUGR, and when the obstetrician was unsure whether to deliver, 98% of these patients (*n* = 376) completed a 2 year follow-up, revealing that the rate of cerebral palsy was greater for patients delivered prior to 31 weeks PCA, and prematurity-related complications were important contributors to this risk (127). Notably, the relationship between gestational age, IUGR and motor deficits suggests the impact of IUGR becomes more apparent with delivery at a later gestation (128). The large prospective EPIPAGE (Etude EPIdémiologique sur les Petits Ages Gestationnels, epidemiologic study of early gestation ages) study (>5,000 births) examined neurological outcomes in school-age children that were born AGA or IUGR (<10th centile for birth weight) at 24–28 weeks or 29–32 weeks PCA, and found similar cognitive deficits in AGA and IUGR infants born at 24–28 weeks PCA, but much less in AGA infants born at 29–32 weeks (129). This clearly indicates the importance of *in utero* brain maturation up to at least 32 weeks PCA. The rate of neurocognitive deficits in the moderately preterm infants with IUGR was around 40% and was identical to the incidence of neurocognitive deficits in extremely preterm infants (129), indicating that the impact of intra-uterine and extra-uterine adverse conditions may be similar on the developing brain in early third trimester. However, a limitation of the EPIPAGE study was stratification of infants by birth weight alone, with no supporting evidence for IUGR.

Consistent with these findings, another study using neuroimaging showed that at 6 years of age, both extremely preterm infants (born before 28 weeks) and moderately preterm IUGR infants (born after 28 weeks), had decreased brain connectivity (measured using MRI fractional anisotropy) when compared with moderately preterm AGA controls, which in turn is associated with poorer socio-cognitive performance (130). IUGR infants born moderately preterm and assessed at term equivalent age also demonstrated reduced cerebral cortex gray matter volume and lower scores in attention-interaction availability, compared to appropriately grown preterm infants (131). Again, these observations support the importance of *in utero* brain maturation.

Interestingly, the rates of several prematurity-associated neonatal diseases in IUGR vs. AGA infants also vary with the gestational age at birth. In preterm infants born at or before 28 weeks PCA, the rates of IVH, respiratory distress syndrome and necrotizing enterocolitis are largely unaffected by IUGR. From then on, all adverse outcomes including IVH increase in IUGR compared with AGA premature infants, suggesting a need for closer surveillance for IUGR in the moderate and late preterm infants (118, 132). Such findings are of concern since late preterm births account for the vast majority of preterm births. Studies on infants born at later preterm to term gestational ages suggest that impaired fetal growth increases the risk for low intellectual performance (16, 133, 134). Comparing monozygotic twin pairs born after 32 weeks PCA, the growth-restricted twin is at increased risk for low cognitive performance at school age or in adulthood compared to the appropriately grown twin (135). In addition, a further study in twins has quantified the effects of low birth weight, showing that a 500 g increase in (term) birth weight results in a 2% increase in total brain volume, gray matter volume and white matter volume, and a 2-point increase in IQ (136).

In summary, the gestational age at delivery has a remarkable impact for IUGR infants who are born extremely preterm, such that the prematurity-related complications “override” the effects of IUGR on neurodevelopmental outcome. For the moderately late preterm infants, the independent impacts of IUGR and prematurity on neurodevelopmental outcome becomes more apparent. Altogether, these studies highlight that we need more information on when to deliver IUGR babies, and the criteria on which to base this decision.

## Clinical Trials and Presumptive Therapies

We wish to acknowledge the enormous contributions of researchers and clinicians in bringing therapies to clinical trials and generating the preclinical data to support these transitions, although the purpose of this article is not to review all of these. We will highlight some trials and their outcomes as, while we have no conclusively effective therapies as yet, we can learn a great deal about improving patient stratification and the efficacy in drugs with shared mechanisms of actions. For example, a recent double-blind randomized study using dydrogesterone, a synthetic progestogen, has reported increased birth weight, and decreased MCA resistance index in idiopathic IUGR (137), supporting the therapeutic use of progesterone replacement suggested in preclinical studies in guinea pigs (138). Specifically, in this human trial weight increased by ~50% in the treatment arm, vs. 23% in the control arm. Although these effects are promising, this was a single center, small study (89 participants), recruiting early and late IUGR (range 28–35 weeks of gestation) with no postnatal follow-up, thus further work remains to be done. The action of dydrogesterone includes effects that altogether increase myometrial perfusion and immunomodulation by increasing progesterone-induced blocking factor (PIBF) levels (139). PIBF is secreted by peripheral lymphocytes from healthy pregnant women, and it has important immunomodulatory functions that appear to protect fetuses from resorption and therefore plays a role in the maintenance of pregnancy, most likely by inhibiting NK lymphocytes and producing a dominant TH_2_ cytokine response (140); hence, many poor pregnancy outcomes including miscarriage and preterm birth are linked to low PIBF levels. Manipulation and control of PIGF levels in pregnancy is therefore a priority for researchers.

The STRIDER-UK study (multicentre, randomized, double-blind, 156 participants) tested the use of sildenafil in women with severe early-onset IUGR and found that treatment did not prolong pregnancy or cause any adverse effects, but did not improve pregnancy outcomes (141). However, this study is part of a more extensive international study, and in isolation, it does not have the power to adequately assess outcomes in this very high-risk cohort (45% of recruited infants in this study died) (142). A meta-analysis (nine studies, total of 576 treated patients) has shown that arginine supplementation increases gestational length and birth weight in IUGR pregnancies, except for infants born preterm (<32 weeks PCA) with severe IUGR (143); there were no reported side effects. The proposed mechanisms of action of arginine include the increased production of placental insulin that acts as a fetal trophic factor.

Creatine may also be a potential treatment for IUGR (144, 145), with recent studies showing a positive correlation of birth weight to placental creatine load (146), and the discovery that the human placenta expresses the enzymes to synthesize and transport creatine (147). Creatine is an energy substrate which protects ATP turnover during periods of oxidative stress (148) and as such may be a potential prophylactic treatment for IUGR outcomes (144, 145). Creatine readily crosses the placenta in humans and some other omnivores (but not in sheep, an herbivore), suggesting that maternal creatine supplementation could be used to increase placental creatine transfer and promote fetal growth in a hypoxic uterine environment. Supporting data include a number of pre-clinical studies (149–151). Whilst there has been extensive animal research suggesting creatine's potential to protect the fetus against periods of oxygen deprivation in several animal models, and there is a strong rationale for moving toward clinical trials for maternal creatine supplementation to reduce or prevent IUGR, no intervention studies have yet been undertaken in pregnant women. IUGR is also proposed to be a disorder of insulin-like growth factor-1 (IGF-1) deprivation (discussed below). Of particular note is an extensive preclinical study of prenatal IGF-1 treatment in a sheep model of IUGR (41 controls, 66 IUGR + saline, 28 IUGR + IGF-1, powered for sex-specific analysis and long term follow-up) which show that prenatal IGF-1 treatment improves prenatal and postnatal indices of growth and biochemical dysfunction in IUGR (152, 153).

## Overcoming the Hurdles to Progress

### Early Markers for IUGR Screening: A Challenge Faced by Researchers and Clinicians

Less than 30% of infants with a birth weight <10th percentile are detected during pregnancy (154, 155). Infants born after undiagnosed IUGR have 2–9 times higher risk of perinatal death and severe neurological complications to those diagnosed prenatally (156–158).

The current, commonly used antenatal assessment of IUGR is examination of the symphysiofundal height, and this has a sensitivity of just 17% and a positive predictive value of 20% (159). Once a red flag has been raised, even selective ultrasound and universal ultrasound perform poorly with a sensitively of 20 and 57%, respectively (160). This perhaps reflects the larger caliber vessels and reduced downstream resistance of the late preterm placenta that means that the umbilical artery Doppler parameters are rarely abnormal; indeed, most adverse events in late pregnancy occur in fetuses with normal Doppler readings (161). However, as mentioned above, FGR babies assessed at term age will often demonstrate abnormal middle cerebral artery (MCA) blood flow represented by a reduced MCA pulsatility index (PI) due to “brain sparing.” A more sensitive and specific measure of fetal well-being may be the cerebral-placental PI ratio, which is calculated as the MCA PI divided by umbilical artery PI. This parameter has improved the sensitivity for detecting babies at risk of adverse perinatal outcome including perinatal mortality, admission to NICU, low 5-min Apgar score and cesarean for fetal distress (26, 162, 163).

Given the importance of placental insufficiency for the origin of IUGR, blood-based markers in the mother that relate to early-onset placental insufficiency are logical starting points for identifying biomarkers that detect IUGR. Biomarkers are molecules, genes, or a particular combination of these by which a pathological or physiological process is identified. Recent reports of biomarkers in maternal blood related to early-onset placental insufficiency include pregnancy-associated plasma protein-A (PAPP-A), alpha-fetoprotein (AFP), inhibin A, placental growth factor (PlGF), uric acid, and free beta- or total human chorionic gonadotropin (102, 164). For example, a 2 to 3-fold increase in late-onset IUGR is noted in women who were found to have elevated first trimester PAPP-A and elevated second trimester AFP (165). Combining maternal risk factors, biochemical markers such as ADAM12 (A Disintegrin and Metalloprotease-12) and placental protein-13, plus abnormal uterine artery waveforms for the prediction of late-onset IUGR (with a false positive rate of 5%), provides a sensitivity of 61% for IUGR at <37 weeks but unfortunately only 32% for IUGR at >37 weeks (166).

The use of these putative biomarkers (and the development of new biomarkers) is constrained by the absence of robust baseline data on their expression and function. We could overcome these problems with population profiling and further basic research into the mechanisms underlying typical placental development and function. Biomarkers for identification and stratification could improve outcomes even with current clinical practices, and are likely to have two additional benefits: firstly, prenatal treatment options [e.g., intra-amniotic IGF-1–(153)] are showing great promise, and biomarkers would identify those needing therapy; and secondly, by avoiding treatment of healthy babies and the risk of complications arising from newly developed prenatal therapies.

*In utero* assessment of brain structure and metabolism is now possible, and these techniques have been applied to cohorts of IUGR infants (167). These studies aim to understand the underlying pathology, but also to determine biomarkers as indicators of pathology and outcome. For example, in 19 IUGR and 25 non-complicated pregnancies, T2-weighted MRI allowed for the assessment of neuropathology and magnetic resonance spectroscopy allowed for analysis of indices of cell membrane and myelin formation (choline), glycolytic enzyme activity as an indicator of cerebral hypoxia (lactate), and for cerebral mitochondrial function (NAA) (167). Of the 15 infants with complete data sets in the IUGR arm, three died *in utero*, and only two had an uncomplicated neonatal course. However, MRI was not sensitive enough to detect injury in any of the 15 IUGR infants *before delivery* (median scan age of 27 + 6 weeks PCA). Interestingly, fetal brain lactate levels were elevated in three control infants with normal progress of growth, birth and outcomes. Increased brain lactate has previously been considered a hallmark of hypoxic changes, but lactate may also play a role as an energy source in the developing brain (168). The authors suggest that the altered magnetic resonance spectroscopy parameters represent changes in mitochondrial metabolic status, and these warrants further study in a preclinical model as a novel therapeutic approach. This study is a valuable example of how these still tricky and expensive prenatal screening techniques could be applied to larger cohorts, but whether this effort can provide invaluable data on the mechanism of damage and criteria for risk stratification will require further research.

Circulatory biomarkers based on the detection of microRNAs (miRs), mitochondrial DNA (mDNA), cell-free RNA (cfRNA), and exosomes could also have diagnostic value, but remain to be fully validated. Two recent studies demonstrate how these new analytic approaches provide early diagnostic biomarkers for preterm birth and preeclampsia. Firstly (169), demonstrated that a panel of seven cfRNAs present in the maternal blood had utility in predicting preterm birth with an AUC (area under the curve) of 0.81. Similarly, Jelliffe-Pawlowski et al. (170) used a 25-target screen of serum proteins that, together with maternal risk factors, predicted preterm birth with an AUC of 0.806. Both studies might be said to be limited by low patient numbers and a lack of ethnic diversity in the patient groups, but this multi-marker approach seems to increase sensitivity and specificity compared to previous mono-marker approaches, and improvements (decrease) in the time needed to perform such analyses means that effective bedside screening is becoming a reality.

A further elaboration on the multi-marker approach is the application of personalized risk-based screening methods that combines maternal factors and biomarkers, as in the combined use of uterine artery Doppler, maternal risk factors, and serum biomarkers. This approach, taken as part of a study nested in clinical trial for pre-term preeclampsia (ASPRE, Aspirin for Evidence-Based Preeclampsia Prevention) (171), showed clearly that prospective screening for preterm preeclampsia by means of the FMF (Fetal Medicine Foundation) algorithm, which combines maternal factors and biomarkers at 11–13 weeks' gestation, was better at predicting disease risk than current biometric criteria.

### A Holistic vs. Mechanistic View of Modeling IUGR—How to Improve the Validity of Our Models

An important question when modeling any disease or disorder is whether it is necessary for the phenotype (in this case a reduction in body and brain weight) to be the same as observed clinically if we don't know that the mechanism of injury has been faithfully reproduced. Also, how much importance should we place on the phenotype (body and brain weight) being matched if we have to apply an injury/perturbation (in type or magnitude) that is not clinically relevant? In the case of IUGR there has been a proliferation of animal models that produce fetal growth restriction, but often by means that have limited clinical reality; e.g., abrupt and late onset reductions of uterine or umbilical blood flows. These procedures do produce hypoxia-induced cell death, and various complex inflammatory processes that help us to understand and look for effects in IUGR infants, but they are of limited use in understanding how IUGR and placental dysfunction actually arise in human pregnancy. Perhaps, with our increasingly detailed knowledge of how and when placental dysfunction in IUGR occurs (88, 172, 173), we could aim to model these specific changes, and this would be valuable in the collective move toward developing therapies that can be applied early in pregnancy. We will below describe some of the common approaches to modeling IUGR and some suggestions for how these could be altered to add to our collective data on how to prevent IUGR, and treat or repair the damage caused by IUGR.

*Surgical interventions that reduce perfusion of the maternal or fetal side of the placenta:* The seminal study demonstrating that poor placental growth itself causes attrition of fetal growth is that of Wigglesworth (174). Since that time there has been a proliferation of models of IUGR, predominantly focused on fetal/neonatal weight and based on the conceptual paradigm that it is utero-placental perfusion that determines placental function, which in turn determines fetal growth and the vulnerability of the fetal brain to damage. These models of IUGR include (but are not limited to): acute onset hypoxia/ischemia (uterine artery ligation), progressive onset hypoxia/ischemia (uterine artery restriction); and placental damage/reduction (inert microsphere injection, partial placentotomy). However, these approaches offers little consideration to the idea that the fetus(es), or the feto-placental compartment might themselves be the source of physiological changes that cause the typical uterine hyperaemia and the appropriate increase of utero-placental perfusion with increasing gestation. Evidence of this type (and almost forgotten) was acquired nearly 50 years ago by Christopher Bell in guinea pigs who showed that progesterone produced by the placenta causes a loss of constrictor adrenergic nerves in uterine blood vessels, and simultaneously induces synthesis of a vasodilator mechanism not present in the non-pregnant uterine vasculature (175, 176). Hence, there is a good reason to think that a cause of IUGR is the *failure* of the placenta to adequately modify the uterine circulation to support time-dependent fetal growth. As such, more work needs to be done on the differences in feto-placental signaling as a driver for poor utero-placental vascular development across gestation.

In all experimental studies to date, the attention is almost always directed to the fetal effects, with few observations made of maternal or placental physiology, especially the clinical conditions usually associated with IUGR, such as uterine artery and myometrial remodeling, immune regulation of trophoblast implantation, placental metabolism *per se*, and the causes of placenta infarction and abruption, etc. A chief mechanism by which we should validate animal models of IUGR should be to measure uterine blood flow, as is done clinically. Admittedly, historically this was not easy to do in rodents, although it is increasingly possible due to advancements such as ultrafast doppler. We now have the ability to measure these clinical indices and to associate them with fetal outcomes as we attempt to better understand this relationship.

*Poor placental structure and function—from the beginning:* Placental insufficiency is a process that evidence suggests is present from the earliest stages of trophoblast invasion, supported by the observation of abnormal levels of inflammatory and placental factors from the 1st trimester with poor pregnancy outcomes [(47) and reviewed in Kane et al. (177)]. From the time of trophoblast invasion into the endometrium, the uterus undergoes phenomenal modification (178, 179), increasing in weight and internal volume by ~16- and 500-fold, respectively, together with major re-modeling of the uterine vasculature (as discussed above) to create a low resistance, non-reactive vascular bed. It is perhaps not surprising that these features have received less attention as determinants of IUGR because, except for non-human primates, these key features of pregnancy are not present in the commonly used laboratory animals. However, comparative studies of more unusual species, like the Spiny Mouse (*Accomys caihrinus*) reveal that unlike in the conventional rodent that there is a large vascular contribution to the fetal membranes originating from the umbilical vessels close to the fetal surface of the placenta (unpublished observations). As such, studying basic process of placentation in small species such as these may be a useful start to developing models of early-onset placental dysfunction that mimics what is seen clinically (57, 152, 180). Non-traditional species such as the spiny mouse, gerbil, and guinea pig studied from the time of conception offer advantages such as a relatively long gestation (from 39 to ~67 days), and the birth of offspring where development of the major organ systems is largely complete at the time of birth. For the spiny mouse in particular, an additional advantage is that the fetal adrenal gland produces dehydroepiandosterone and cortisol (181–183), with evidence of the presence of a feto-placental unit as in humans, and not present in other rodent-like animals, or sheep.

A clear example of how a candidate mechanism has been effectively applied to model IUGR is the knockout of IGF2 production in the mouse placenta. Levels of the IGF proteins are often reduced and IGF1 binding proteins increased in the placentas of IUGR human pregnancies (184), although this is not consistently found (185). The regulation of IGF signaling is a complex balance between the IGFs and their binding partners that regulate bioavailability during pregnancy, including in IUGR [reviewed in Martin-Estal et al. (186)]. Mice born from dams with a placenta-specific IGF2 gene knock out (KO) have clearly reduced body weight, brain injury, and lasting cognitive and metabolic phenotypes with postnatal development. The mating of the IGF2 placenta-specific KO mice with the endothelial NOS (eNOS) KO mice provides an interesting example of the cross-talk between (putative) pathological mechanisms. As expected, the phenotype of these two KOs was additive (more severe IUGR), but the diminished placental nutrient transport typically observed in the eNOS KO mice was not seen in the IGF2-eNOS double crossed mice (187). The basis of the application of the eNOS KO mice to IUGR research is that the absence of eNOS reduces the capacity of the maternal vascular to accommodate the changes in blood flow necessary for adequate placentation, and that the dams are hypertensive (including with proteinuria) as observed in women with pre-eclampsia, a risk factor for IUGR. Thus, it was concluded that a “multiplicity of dysfunction” probably underlies IUGR in women, so the multiple facets of vascular dysfunction in this mouse line would be useful to assess placental hypoxia with free radical formation, reduced placental nutrient transport capacity, and reduced fetal growth (188).

Assessment of the epigenetic landscape of the eNOS and IGF2 genes from IUGR placental tissues shows that epigenetic modifications are present and might be drivers of gene dysregulation (189). However IGF2 gene expression changes and methylation changes of IGF2 are not consistently reported in clinical studies, One of the negative clinical studies found neither gene or methylation changes the cohort was older mothers (39–40 years of age), although changes in placental methylation are only detectable with the techniques used when substantial differences are present (173). Alternatively, this negative study is evidence that methylation and gene expression changes in IGF2 are not present in the first trimester but evolve over time, and so this important study that warrants repeating with larger and more diverse cohorts.

*Immunological dysfunction:* There is clear evidence that the immune system in women with an IUGR pregnancy differs compared to those without IUGR (190). This study found that peripheral monocytes in women with an IUGR pregnancy had a more classically anti-inflammatory profile than monocytes from women with an uncompromised pregnancy. In addition, when peripheral blood mononuclear cells from women with IUGR and normal pregnancies were stimulated with trophoblast antigen (191), a greater pro-inflammatory reaction occurred in women with an IUGR pregnancy. When these dysfunctional processes first arise in pregnancy, and their impact on early placentation, would be interesting to pursue further, albeit requiring large cohorts of prospectively recruited, not-yet-pregnant women—a difficult task, but one with important public health outcomes. This information would then be possible to overlay into more specifically focussed animal models.

As pointed out by Sir Peter Medawar many years ago (192) it is clear that immune modulation is a fundamental response to conception that allows implantation and persistence of the fetus as a foreign allograft, and a response likely to be shared by humans and laboratory animals. It is a process that involves a phenotypic transformation of decidual macrophages and natural killer cells that occurs in parallel with remodeling of the vasculature adjacent to the implantation site. As such, it is possible that these shared features could be used to drive spontaneous cases of IUGR in non-human pregnancies. Indeed, some cross-breeding of mice of mixed genetic background has been found to produce IUGR of varying degrees, together with, as for most clinical situations, other complications of implantation, uterine re-modeling, and pathophysiology suggesting preeclampsia in the dam (193, 194). These “immune” models have been shown to have similarities pathological changes as occurs in the human IUGR placenta in the trophoblast remodeling protein Formyl peptide receptor-2 [FPR2; (195)], and a dysfunction of decidual arteriolar remodeling (196) such as that associated with pre-eclampsia and IUGR (197). Thus, we may have experimental models at hand which will aid in the understanding of the origin of IUGR together with the other obstetric problems that usually accompany it. A strength of this approach is that each of the parents and the offspring are immunologically competent, and it is only the combination of the fetal and maternal tissues that is abnormal. This model will require further assessment of fetal brain phenotypes to support the similarities in placental immune dysfunction to the human, but it promises to be valuable to assess therapies to minimize the neonatal morbidities that IUGR produces.

Finally, we would like to highlight the comments made by Saleem et al. from Karachi, Pakistan that “Concerted efforts should be made to gather indigenous data about the risk factors of IUGR that are more pertinent to our population. Evidence-based recommendations deduced from such data sets are more likely to be successful and valid” (198). Although they were addressing the need for improved medical services, we think this comment also points to the need for improvements in preclinical modeling to be “*population specific*.” This specificity must take into account not just high vs. low income, but etiologies and conditions specific to local care centers, and this is where in-depth epidemiology needs to take a more prominent role in guiding preclinical research.

### Human Neuropathological Studies and How They Need to Play a Bigger Role in the Study of IUGR

The emergence of modern medicine was firmly based on pathology and post-mortem examinations, and while never being able to identify mechanisms of disease or injury *per se*, have been invaluable as the basis for setting diagnostic criteria and the effectiveness of treatments and interventions. The expectations of many that modern imaging, genetics, and -omics would make pathology obsolete has been a significant error of twenty-first-century science, and in neonatology it was high-quality neuropathology studies by groups in Portland (OR, USA), Paris (France), and Boston (MA, USA) (199–201) that were persuasive in changing our understanding of the genesis of perinatal brain injury over the past 20 years. Specifically, these foundational studies provide the basis for interpreting MRI studies, and for designing improved animal models to test neuroprotective strategies. This is important because most IUGR babies survive, and the injuries present in very severe IUGR fetuses may not be representative of the major population of IUGR infants.

It is indeed worth highlighting a number of human studies; this is not exhaustive but is intended to allow us to remark on what we have learnt, and what we need to add to the field. Studies from Samuelsen et al. (202) show a reduction in the numbers of cells in the brains of IUGR infants; these infants displayed brain sparing (relative increase in brain weight to body weight) but still had significantly lower brain weights. In this study, control fetuses acquired an average of 173 million cells per day from mid-gestation to term, and the IUGR fetuses acquired only 86 million new cells per day. Samuelsen et al's exhaustive cell counting assessment [reviewed in, Larsen (203)] supports the findings of two studies from over 30 years ago which used a lower total DNA content to infer a reduction in cell numbers (50, 204). Samuelsen's conjectures that his data supports the hypothesis that the reduction in head circumference in IUGR is due to reduced proliferation rather than cell death is provocative, but needs to be confirmed by objective assessment of cell death and proliferation.

Another set of studies includes a concerted effort by researchers based primarily at the Mater Hospitals in Brisbane, Australia, that included 37 asymmetric IUGR cases (weight <3rd centile) identified from a series of 225 stillbirths (205). This first study, over 20 years old now, critically demonstrated the importance of cell death, and to a lesser extent astrogliosis, in the IUGR brain. It is worth noting that although the analysis was limited to hematoxylin and eosin assessment of cell death, and in some cases glial fibrillary acidic protein for astrocytosis, that even with the expertise of a neuropathologist no injury was found in 5 of the 37 brains. Of these 5 “uninjured” IUGR brains, four were from infants below 26 weeks PCA—does this suggest an important window for treatment specificity? Striking limitations of this study are that there was no breakdown of the specific gestational ages of the infants, no body or brain weight data, and no case-by-case description of the findings, making it very difficult to draw further conclusions. These authors overcame many of these limitations in a more recent publication (206) using brains from the first cohorts and sourced from a further 305 stillbirths where they assessed cell death with three separate markers. This more recent study clearly showed that third trimester stillborn fetuses with both IUGR (weight <3rd centile) and placental infarction (<5% of total placental villus surface) had neuronal apoptotic changes in regions including the pons and the frontal and temporal cortex. These studies involved the use of controls that were stillborn but had neither IUGR nor placental infarction. Staining for micro- or macrogliosis or proliferation was not included, unfortunately.

### The Value of Continuous Neuro-Monitoring to Understand Risk for the IUGR Infant

Much has already been reported on the neuroimaging findings of IUGR infants and their relationships to neurodevelopmental delay. However, neuroimaging such as MRI can only be done at specific time-points and is not applicable as continuous neuro-monitoring to reveal temporal changes with impact on clinical conditions and to guide management.

#### Cerebral Haemodynamic Measurements in the IUGR Fetus

With advancing fetal hypoxia and compromise, the cerebral haemodynamic response involves two components—firstly an initial stage of increased cerebral blood flow aimed at protecting the brain (brain sparing), followed by a second decompensatory stage that is associated with brain injury, and probably due to the increased cerebral blood volume (207). At the early stages (i.e., brain sparing) blood flow (CBF) of the frontal lobes is increased, perhaps with the effect of protecting higher cognitive functions, but under chronic and more severe circumstances this change is lost and CBF is diverted to deeper (more essential?) structures such as the basal ganglia and the brainstem (29). Also, in the IUGR fetus there appears to be a loss of cerebral vasoreactivity; using prenatal Doppler sonography, a subset of IUGR fetuses did not show the expected rise in cerebral resistance in response to maternal hyperoxygenation, suggestive of impaired cerebrovascular regulation. Indeed, these “non-responders” had a higher risk of being delivered for fetal distress, indicating that they were more compromised (208).

It is interesting that there is an increased incidence of stroke in adults born with low birth weight. The association between low birth weight and adult stroke was most pronounced for individuals with relatively increased head size, suggestive of *in utero* conditions that induce brain sparing (13). It is very plausible that these mechanisms of increased adult stroke are due to vascular remodeling secondary to the shear stress and wall tension, leading to structural changes in the vascular wall (209). Thus. understanding IUGR-associated cerebral haemodynamic changes has implications for improving brain health even in adults.

#### Cotside Cerebral Haemodynamic Measurements: Cerebral Blood Flow and Oxygenation

Studies in IUGR infants have made the following important observations:

- increased CBF on the first day of life (210);
- reduced cerebrovascular resistance and persistent dilatation of the cerebral arteries (211, 212);
- higher regional cerebral oxygen saturation and reduced cerebral oxygen extraction within the first 24 h (213) and up to 3 days of age (214); and,
- cerebral haemodynamic parameters normalize within a few days of birth (210, 211, 213).

To date there is little postnatal research investigating the relationship between cerebral hemodynamics after birth and neurological injury in IUGR infants; this is an area of need as there is evidence to indicate that the altered cerebral hemodynamics that exist in the IUGR fetus persist postnatally. Notably, cerebral oxygenation has been shown to predict neurodevelopmental outcome in preterm infants (215). Moreover, fluctuations of cerebral oxygenation and oxygen extraction have also been related to the occurrence of intracranial hemorrhage in preterm infants (216, 217). Finally, if CBF remains elevated when the neonate is no longer exposed to a hypoxic environment, the increased CBF could cause hyperoxia within the fragile brain and contribute to further neurological damage (218).

#### Cotside Cerebral Haemodynamic Measurements: Cerebral Autoregulation

Autoregulation is the ability of the cerebral vasculature to maintain reasonably constant CBF despite fluctuations in cerebral perfusion pressure which are mainly affected by changes in systemic blood pressure, and it is likely that autoregulation is impaired in sick preterm neonates and this contributes to the cerebral ischaemic and haemorrhagic injury (219). As IUGR fetuses are often delivered preterm, and due to the vascular structural and functional changes set in place by the “brain sparing” response to IUGR, these infants are theoretically at risk of impaired autoregulation, resulting in cerebral hypo- or hyperperfusion when systemic blood pressure fluctuates. In case of prolonged brain sparing due to chronic hypoxaemia, maximal cerebral vessel dilatation may have already been reached and may persist after birth, which would further limit protective autoregulatory responses. In addition, IUGR neonates appear to have higher blood pressures compared to their AGA peers (30, 220), theoretically contributing to risk of cerebral hyperperfusion and hemorrhage in the presence of a pressure-passive cerebral circulation; indeed, this has been shown in IUGR lambs where, compromised structural integrity of the cerebral microvasculature leading to cerebral hemorrhage has been demonstrated (82). Despite its clinical significance, to date no study has investigated cerebral autoregulation in human IUGR neonates.

#### Cotside Electroencephalographic (EEG) Monitoring

Cerebral electrographic activity can serve as an indicator of neuronal integrity, organization, and the differentiation and maturation of brain networks in term and preterm newborns (221). In acute brain insult, electroencephalographic (EEG) activity shows various degrees of depression, and its severity parallels the magnitude of the brain lesion. These “acute-stage” abnormalities gradually improve with time and are replaced by “chronic-stage” abnormalities such as dysmaturity and disorganization of the EEG pattern (222). In the weeks following preterm birth, IUGR infants reportedly have altered EEG and amplitude integrated EEG, which has been correlated with poor neuromotor development (223, 224). In contrast, another study found that the subset of stable preterm IUGR with good clinical indices had more mature EEG patterns compared to the AGA peers (225), and similarly, preterm IUGR infants had accelerated EEG power spectrum maturation compared to preterm AGA controls at 1 month post-term equivalent age (226). However, no differences were observed at 6 months' post-term age between preterm IUGR and AGA infants, or in comparison to a term AGA group, suggesting such changes may resolve with time (226). Notably, using visual evoked potentials as an indicator of brain myelination in preterm infants born at <33 weeks of gestation age, shorter visual evoked potential latency, suggestive of increased myelination, was found at 6 months post-term age in IUGR infants who had fetal Doppler parameters showing brain sparing (227). The shorter latency was no longer detected at 12 months of post-term age and visual functioning was not affected when followed up at 11 years of age (228). These findings suggested transient accelerated neurophysiological maturation in the IUGR infant brain, possibly as an adaptive process to the severe fetal growth restriction. Overall, it appears there are temporal EEG characteristics in IUGR infants which may relate to their neurodevelopmental outcome. However, EEG data on preterm infants with IUGR are few, as are studies that link EEG parameters with MRI-derived tractography. Early and prolonged continuous recording in larger populations would be required to clarify the prognostic value of EEG in IUGR infants.

## Innovations for Use in IUGR Research:

### Nanomedicine Approaches

Engineered nanomaterials offer therapeutic options as diverse as implantable monitoring devices, drug delivery scaffolds, and wound dressings. An excellent review on nanomaterials for perinatal applications was recently published (229), but we will highlight ways in which these approaches could be employed to improve infant neurological outcomes in the context of IUGR. The greatest need for the IUGR fetus is safe, effective therapies that can be delivered as early as possible in gestation and without the need for preterm delivery. As such, we should turn our attention to nanomedicine approaches if these can be maternally delivered and directly affect the function of the placenta. Nanoparticles delivered intravenously can cross the placenta, and are found in the fetus, including in the non-human primate [see Menezes et al. (230)]. They can also be found in the fetus when administered intranasally to pregnant rats (231). A recent study monitored the “protein corona” of polystyrene nanoparticles, which is the spontaneously adsorbed protein on the nanoparticle surface when the nanoparticles were exposed to maternal plasma. They found that the protein corona formed by maternal serum included vesicular transport proteins such as clathrin, tubulin, actin, and Ras. As such, rather than pose any barrier to transmigration, maternal serum proteins increase the transport of the polystyrene nanoparticles across the *ex vivo* placenta (232). However, in the pregnant rat, inhaled silver nanoparticles caused increased fetal resorption and increased expression of placental inflammatory markers (231). Although this silver nanoparticle study suggests caution in the application of nanoparticles, we can overcome these problems via our ability to modulate the size, physical composition, charge, and delivery method of nanoparticles. Of note, another recent study has shown success in reducing fetal inflammation and neonatal brain injury in a mouse model which mimics exposure to inflammation in the preterm infant. Specifically, polyamidoamine dendrimers were used to carry the anti-oxidant n-acetylcysteine into the blood of the dam (233). This dendrimer treatment was associated with improvements in inflammatory and neuropathology indices in the fetal compartments. It is important to note that these polyamidoamine dendrimers did not need to pass to the fetus to be neuroprotective and thus, this maternal-placental delivery reduces any potential of off-target, damaging effects of stray nanoparticles in the fetus.

Work from our lab has taken the approach of targeting one cell type—microglia—rather than being compartment-specific (234). Microglia are regularly found to mediate injury in neurological paradigms. The microglia-specific nanoparticles in our work are comprised of DNA, further demonstrating the flexibility in the design possibilities of nanoparticles. However, we do not yet know if these DNA nanoparticles cross the placenta, and what specific aspect of microglial activation would be the most appropriate to target in IUGR. There is a lack of knowledge of nanoparticle biodistribution in non-human primates, or other species with more human-like placental structures, such as the spiny mouse (235). More studies using IUGR models developed in species with a more human-like placental structure would be valuable steps toward uncovering the potential of nanoparticles to deliver anti-oxidants, vasodilators, or growth factors to specifically target pathological processes in the placenta.

Altogether these tools and approaches show that we have made the technological advances and have the monitoring skills with which to move forward to design targeted therapies for the placenta and the fetus to overcome the damage associated with IUGR.

### Using Advanced Imaging and Histology Techniques

We have a poor understanding of the direct correspondence between medical imaging outputs and tissue microstructure. This lack of knowledge is due to differences in scale and resolution between medical imaging modalities and traditional neuropathology techniques that have been further frustrated with complexities of image registration (236). A lack of knowledge of the actual biological substrate of imaging outputs frustrates our ability to understand the specific nature of changes occurring in the IUGR infant brain, as the most severely affected infants do not survive, as discussed above. Improvements in histological techniques, specifically the use of optically clear histology, is allowing us to overcome some of these hurdles (237), but obviously not in living tissue. Optically clear histology allows whole brains from small experimental animals, and sections of human post-mortem tissue, to be made optically clear and stained with multiple specific markers of various cell types. This advance allows us to visualize the 3D organization of tissue microstructure in tissue sections large enough to be studied beforehand with MRI techniques, and then to co-register the imaging and histological data. This approach has been applied to the developing mouse brain to provide data on what types of cells or cell compartments contribute to specific imaging signals (238). A finding from this study worth noting is a positive correlation between mean MRI diffusivity and cell density, which is unexpected based on the current assumption that cells provide an impediment to the diffusion of water molecules in tissue (238). This and other findings suggest that a great deal of work in human post-mortem tissues is required to ensure that our assumptions of the microstructural correlates of human imaging in the literature are robust (239, 240).

The current gold standard for functional brain imaging is blood–oxygen dependent (BOLD) signals measured using MRI (241), but this technique is costly and practically difficult to use in infants. An easier to implement optical imaging alternative is functional near-infrared spectroscopy (fNIRS), which also measures the level of blood oxygenation and can be applied on neonates or young children through the skull but with spatial resolution measured in mm (242). The next level of technological development is functional ultrasound (fUS) neuroimaging that provides high sensitivity imaging, with a resolution of ~100 μm, and can identify cerebral blood volume changes in the whole brain without contrast agents. The fUS image is based on the phenomenon of neurovascular coupling, about which little is known in the developing brain [see, (243, 244) for recent animal studies] and the ability to measure cerebral blood volume changes with high sensitivity, which is an attribute of fUS (245). Non-invasive fUS imaging of brain activity in humans is possible through the fontanel of human neonates at the bedside. In combination with surface EEG recordings in preterm babies, fUS allowed for the estimation of cerebral blood volume variations to measure the spatiotemporal dynamics of epileptic seizures (246). The sensitivity of fUS, such as measuring small diameter blood flow even in rodents with millisecond temporal resolution, should allow it to become a useful and valuable tool to understand the effects of IUGR on the brain in clinical and in preclinical settings. We have applied fUS to study the brains of rats in a paradigm of protein restriction-induced IUGR and found that it identified connectivity deficits (59), and was able to validate improvements in connectivity associated with neuroprotection. For further comprehensive information on the applications of fUS, please see the recent review by Deffieux et al. (247).

### Genomic Analysis as a Means to Diagnose, Stratify Risk and Understand Disease Mechanisms

Genomic screening offers the promise of early and specific diagnosis, risk stratification and personalized therapeutic deployment. There are inherent ethical issues with genetic screening that are in no way limited to the field of IUGR. These include incidental findings of unknown significance and the more complex issue of the rights of the child, and specifically the child's right “not to know.” Violations of the child's rights may relate to the discovery of gene variants that have no bearing on fetal development or childhood health, but which alter adult disease risk. Although generally these problems are overcome with targeted analysis, variants with impact at multiple stages of life make this problem harder to manage. For a lively discussion on bioethics related to prenatal genome sequencing we refer the reader to these references (248–250).

Despite these concerns, non-invasive prenatal screening (NIPS) for fetal aneuploidies using cell-free DNA has been widely adopted in clinical practice due to its improved accuracy compared to traditional screening approaches (251). NIPS approaches can include whole genome sequencing techniques with high sensitivity and specificity even with very low sample input (252). The benefits of whole-genome sequencing include the identification of micro-deletions that would be missed by karyotyping. These micro-deletions are relevant to human growth and have identified some genetic causes of IUGR (253). It might not be necessary to sequence the genome to identify disease risk but to examine single nucleotide polymorphisms (SNPs). SNP analysis is increasingly accessible with sequencing, and whole-genome SNP arrays can be processed within 3 days (254). This short time frame means that the data can affect prenatal diagnostic decisions, and in the future, may guide treatment decisions. An approach to risk stratification has been tested in the related field of preterm birth, identifying an unbiased association of SNPs with white matter imaging phenotype. This approach has successfully identified several SNPs that associate with a clinical white matter phenotype in preterm infants—namely, specific SNPs within the genes for *FADS2* (Fatty Acid Desaturase 2) (255), *PPARG* (Peroxisome proliferator-activated receptor gamma) (256), and *DLG4* (discs large homolog 4, the gene Post-Synaptic Protein-95) (257). Although these SNPs provide a biomarker for infant outcome, they are also under further investigation to understand the mechanistic link between these genes variants and brain development.

What is lacking in the field of IUGR to date is the application of these genomics approaches in massive populations to identify variants denoting risk of obstetric and (neuro)developmental outcomes. In the fields of Alzheimer's disease and cancer, it has taken the study of many thousands of patients (and their tumors) to identify risk phenotypes that are now used to drive drug discovery efforts and to identify targets for personalized drug therapy, and these approaches have been effective in public health (258). Altogether, dramatic improvements in technology open avenues for understanding three important aspects of IUGR—diagnosis, risk stratification, and mechanisms of disease. The logistical and monetary support to bring together a vast number of patients across multiple centers, and the bioinformatics needed to understand these processes, are now within reach if we apply the lessons from genetics studies of adult diseases.

## Conclusion

IUGR remains a complex health care issue across the globe. Concerted research and clinical effort have raised the profile of this complex problem and improved our knowledge of the neurodevelopmental sequelae of IUGR. What we cannot yet offer in the field of IUGR is an animal model that recapitulates the underlying pathophysiology of IUGR, primarily because we do not yet know the etiology (probably, more than one etiology) underlying the various sub-groups of IUGR. Valuable post-mortem studies, imaging studies using newly optimized *in utero* fetal and placental imaging, and pooling our research data across models to find common elements, will be key to overcoming this hurdle. Optimized fetal and placental imaging will also be important future methods of early diagnosis and risk stratification. These will enable us to treat the IUGR infant *in utero* with the goal of preventing brain injury rather than attempting postnatal repair or regeneration. We do not yet understand the role of neuroinflammation in the human IUGR infant, and as such cannot determine if the multitude of immunomodulatory drugs being fast-tracked in fields such as multiple sclerosis and Alzheimer's disease apply to the IUGR infant. The role of neuroinflammation can be studied by bringing together multiple models capturing many facets of IUGR-related neurodevelopmental injury, and more post-mortem studies. We are also limited in our ability to target drugs to either the placenta or the fetus, but these issues will be overcome by advances in materials bioengineering, and proof-of-concept drugs and tools entering the fields of adult medicine. Given the vast numbers of infants born IUGR due to preventable causes such as malnutrition, malaria, HIV infection, and even psychosocial stress such as domestic abuse, we already have a mandate to reduce its impact across the world. Initiatives such as the Millennium Development Goals are making significant improvements in these areas but require our further support.

## Author Contributions

All authors listed have made a substantial, direct and intellectual contribution to the work, and approved it for publication.

### Conflict of Interest Statement

The authors declare that the research was conducted in the absence of any commercial or financial relationships that could be construed as a potential conflict of interest.
